# Physical fitness and its correlation with handgrip strength in active community-dwelling older adults

**DOI:** 10.1038/s41598-022-21736-w

**Published:** 2022-10-14

**Authors:** Po-Jung Pan, Nai-Wei Hsu, Meng-Jer Lee, You-Yuan Lin, Chih-Chun Tsai, Wang-Sheng Lin

**Affiliations:** 1 Department of Physical Medicine & Rehabilitation, National Yang Ming Chiao Tung University Hospital, Yilan, Taiwan; 2 School of Medicine, National Yang Ming Chiao Tung University, Taipei, Taiwan; 3 Center of Community Medicine, National Yang Ming Chiao Tung University Hospital, Yilan, Taiwan; 4 Public Health Bureau, Yilan County, Taiwan; 5 Community Medicine Research Center & Institute of Public Health, School of Medicine, National Yang Ming Chiao Tung University, Taipei, Taiwan; 6 Department of Mathematics, Tamkang University, Taipei, Taiwan; 7 Department of Physical Medicine & Rehabilitation, Taipei Veterans General Hospital, Yuan-Shan/Su-Ao Branch, Yilan, Taiwan

**Keywords:** Health care, Medical research

## Abstract

In this cross-sectional study, we assessed the physical fitness levels of active community-dwelling older adults. Moreover, we investigated the correlation of their (stratified by age and sex) fitness levels with handgrip strength (HGS). Comprehensive physical fitness tests, including sarcopenia screening, were conducted with a total of 2,130 older adults residing in a rural area of Taiwan. The 5th, 10th, 25th, 50th, 75th, 90th, and 95th percentiles of age- and sex-specific physical fitness levels were determined. Furthermore, we identified the key parameters for assessing the physical fitness of older adults and performed stepwise multiple linear regression analysis. Both men and women exhibited age-related decreases in all aspects of functional fitness, a trend indicating that older adults in Taiwan may lose their independence in the future. Furthermore, the regression analysis revealed that HGS was positively correlated with sex, body mass index, and the results of 30-s arm curl and back scratch tests but negatively correlated with age and the result of 8-foot up-and-go test. Our reference values for physical fitness may help assess the fitness levels of active community-dwelling older adults and design community-based health programs to prevent the early loss of independence in community-dwelling older adults in Taiwan.

## Introduction

Community-dwelling older adults experience several health challenges, such as frailty, osteoporosis, sarcopenia, and various musculoskeletal ailments^1–3^. In a study including a total of 740 community-dwelling older adults, 7.4% of the participants had frailty and 49.7% had prefrailty^1^. In the aforementioned study, approximately 33.3% frail adults had deficits in their activities of daily living and approximately 80% had various comorbidities^1^. In Taiwan, frailty and prefrailty have been associated with recent falls, hip fractures, and osteoporosis in community-dwelling adults aged ≥ 50 years^4^. Moreover, patients with musculoskeletal disorders experience higher levels of muscular deconditioning than do healthy individuals^5,6^ and are less likely to meet the required physical activity standards. A 3-year-long prospective cohort study including community-dwelling older adults in Taiwan revealed that 11.0% of the participants exhibited the gradual impairment of their activities of daily living throughout the study period; age was found to be the most relevant predictive factor for chronic disability^7^. In addition to medical treatment, the importance of preventing and delaying disability cannot be overemphasized. In 2015, a program based on the National Ten-Year Long-Term Care Plan 2.0 was launched in Taiwan to prevent and delay disability in older adults. To maintain their fitness level, older adults can participate in various health promotion activities within their communities, such as exercise or dance programs at long-term community care centers. Thus, strategies to evaluate the physical status of older adults and the efficacy of community-based health promotion programs must be explored.

Physical fitness is defined as the capacity to perform daily physical activities safely and independently without fatigue^8,9^. The predominant forms of physical fitness are health-related physical fitness and skill-related physical fitness. The former is associated with everyday activities, whereas the latter is associated with athletic activities. Rikli and Jones designed a functional fitness assessment tool for community-dwelling older adults; the validated tool comprises multiple simple but effective tests to evaluate the muscle strength, flexibility, aerobic endurance, agility, and dynamic balance of older adults through daily activities^10^. Suitable physical fitness tests can be used to objectively evaluate physical function in older adults who participate in community activities to maintain or promote health^11^. In addition, these tests are widely available, less time-consuming, and convenient and require no special equipment, all of which contribute to the clinical practicality of these tests^12^. Notwithstanding the low precision and specificity compared with advanced laboratory-based assessments, the tests used to assess physical performance in older adults may help evaluate muscular strength and endurance^13–16^, flexibility^17^, cardiorespiratory endurance^18–21^, static and dynamic balance.

Reference values for physical fitness are required to improve the interpretability and utility of physical fitness test results in a clinical setting. Although reference values and equations for physical fitness tests, such as grip strength^22–26^ and the 30-s chair stand test^27^, have been determined previously, these values were determined by considering the results of various trials and merely one aspect of physical fitness. Reference values for the physical performance levels of older adults have been reported in studies conducted in the United States^27^, Brazil^28^, Spain^29^, India^30^, Nepal^31^, and Poland^32^. Although studies conducted in Taiwan have reported ratings to assess the physical performance levels of older adults^33,34^, the studies did not explore the active community-dwellings or considered all aspects of physical fitness or the screening for sarcopenia or osteoporosis. To design tailored programs for active community-dwelling older adults on the basis of their physical fitness level and overall health status, all aspects of physical fitness must be assessed in addition to percentile distribution to help older adults compare their functional status with relevant standards. Furthermore, because the aforementioned study was conducted > 10 years ago^33^, updated reference values for physical fitness must be determined, particularly for active community-dwelling older adults.

Handgrip strength (HGS), a common physical fitness test, is convenient for use in community-based assessments. The decline in HGS is reportedly associated with physical limitations in older adults aged ≥ 60 years^35^. A pooled analysis including a total of 6,426 community-dwelling older adults indicated that low HGS is associated with mortality^36^. Thus, HGS can be regarded as a clinically important indicator of the health status of older adults. Therefore, to simplify the assessment work, HGS test can be used as a single test to efficiently determine the physical fitness levels of community-dwelling older adults and obtain objective data. Hence, in the present study, we investigated the possible correlation of HGS with various parameters of physical fitness.

With the aging of community-dwelling older adults, the annual health expenditure of the government increases every year. Thus, various community-based health promotion programs have been launched for maintaining physical fitness levels and delaying disability in community-dwelling older adults. For clinical purposes, a comprehensive survey encompassing all aspects of physical fitness must be conducted to determine the reference values for the physical fitness levels of older men and women. Thus, in the present cross-sectional study, we sought to determine age- and sex-specific reference values for the physical fitness levels of active community-dwelling older adults and investigate the possible correlation of HGS with the physical fitness parameters of older adults.

## Participants and methods

### Study design and participants

In this cross-sectional study, we reviewed the data of community-dwelling older adults who participated in a community health promotion program run by the Center of Community Medicine at National Yang Ming Chiao Tung University Hospital, Taiwan, between July 10, 2017, and December 25, 2019. During recruitment, experienced medical professionals (P-JP and M-JL) evaluated the health status of the participants. All eligible community-dwelling older adults were subjected to initial screening. The inclusion criteria were as follows: residence in Taiwan’s Yilan County, age ≥ 65 years, desire to undergo evaluation for physical fitness, no major medical concerns, and an ability to perform study-related tasks. Older adults with severe neurological or cognitive impairment, a history of recent traumatic events, acute illnesses such as sepsis, unstable vital signs, or complete dependence on caregivers for the activities of daily living were excluded from this study. All procedures performed in this study were in accordance with the ethical principles of the World Medical Association Declaration of Helsinki and National Yang Ming Chiao Tung University Hospital. This study was reviewed and approved by the Human Subject Research Ethics Committee of National Yang Ming Chiao Tung University Hospital (approval number, 2020A012). Written informed consent for participation was obtained from all participants.

### Data collection

Eligible older adults were invited to our health promotion center at National Yang Ming Chiao Tung University Hospital. The study procedure was divided into the following five steps:

1. Interviewing participants to obtain demographic data
2. Administering a frailty questionnaire: a modified questionnaire from short version of the International Physical Activity Questionnaire (IPAQ)^37^
3. Conducting physical fitness tests to evaluate the participants’ body composition (body mass index [BMI]), muscular strength and endurance (30-s arm curl and 30-s chair stand tests)^38^, flexibility (back scratch and chair sit-and-reach tests; negative score if the fingertips do not overlap or pass the 0 mark)^38^, aerobic endurance (2-min step test)^39^, and balance (single-leg standing^40^ and 8-foot up-and-go^38^ tests); the tests were conducted in accordance with the guidelines of the Sports Department of the Ministry of Education in Taiwan.
4. Screening for sarcopenia with measurements of HGS, calf circumference, and 6-m walking speed^41^
5. Measuring bone density

In the waiting area of the aforementioned health promotion center, a face-to-face interview was conducted with the participants to obtain data regarding their demographics and a frailty questionnaire was administered. Subsequently, all participants were subjected to physical fitness assessment and sarcopenia and osteoporosis screening at a sports ground; the sensor-assistive system (SHM; Acutek) and bone density measurement were located there.

### Outcome measures

#### Demographics

Well-trained researchers collected data regarding participant demographics (e.g., age, sex, and exercise habits) during the interview.

#### Physical activity levels

The aforementioned frailty questionnaire was used to collect data regarding the participants’ HGS, energy level, waking speed, physical activity level, and unintentional weight loss^37^. To evaluate their physical activity levels, the participants were interviewed; data regarding their calorie consumption was collected using the modified short version of the IPAQ (Taiwan version)^37^.

#### Physical fitness levels and sarcopenia and osteoporosis

Trained hospital volunteers assisted a qualified physical therapist (Y-YL) and a physical fitness coach in data collection. Intra- and interrater variabilities were not assessed in this study. Nevertheless, the qualified physical therapist (Y-YL) completed official training and qualified assessments on standard operating procedures. Training sessions were conducted before the health promotion program to ensure that the researchers can duly conduct all study tasks. Furthermore, the aforementioned sensor-assistive system for assessing geriatric physical fitness levels was used in our study to improve test efficiency and precision; this system has also been used previously^42^. We measured the participants’ height and weight (InBody 570; InBody), calf circumference, and bone density (Pegasus; Medilink) and conducted the following tests: 30-s arm curl (SHM16; Acutek), back scratch, 30-s chair stand (SHM12; Acutek), chair sit-and-reach (SHM11; Acutek), single-leg standing (SHM 15; Acutek), 8-foot up-and-go (SHM13; Acutek), 2-min step (SHM14; Acutek), HGS (TTM Digital Hand Grip Dynamometer; TTM110D), and 6-m walking speed tests.

#### HGS

The participants’ HGS was measured twice, and the higher of the two obtained values was recorded. On the basis of the Asian Working Group for Sarcopenia 2019 consensus, low muscle strength was defined as an HGS of < 28 kg for men and < 18 kg for women^41^.

#### BMI

In the present study, the participants were categorized as underweight (BMI < 18.5 kg/m^2^), normal weight (BMI, 18.5–24 kg/m^2^), overweight (BMI, 24–27 kg/m^2^), and obese (BMI > 27 kg/m^2^) on the basis of guidelines of the Taiwanese Ministry of Health and Welfare^43^.

#### Osteoporosis

The T-score is a standard score used for bone density measurements with respect to the mean bone density of a population of 30-year-old healthy individuals. Individuals with a T-score of + 1 to − 1 are regarded as healthy. A T-score between − 1 and − 2.5 indicates low bone density, whereas a T-score of less than − 2.5 suggests osteoporosis. A highly negative value of the T-score indicates severe osteoporosis.

### Statistical analysis

Descriptive statistics, including mean, standard deviation, and percentile (5th, 10th, 25th, 50th, 75th, 90th, and 95th), were used to describe the demographics and physical fitness levels of the participants. The data were stratified by age (groups: 65–69, 70–74, 75–79, 80–84, 85–89, and ≥ 90 years) and sex (men and women). For the community-dwelling older adults, a stepwise multiple linear regression model (entry probability = 0.05; removal probability = 0.10) was constructed for the predictive analysis of HGS (kg; response variable) on the basis of age; sex (men, 1; women, 0); BMI; calf circumference; bone density; physical activity levels; and 30-s arm curl, 8-foot up-and-go, back scratch, single-leg standing, chair sit-and-reach, 30-s chair stand, 2-min step, and timed 6-m walking speed tests. Separate regression models were used for men and women. Moreover, the correlations between age and various physical fitness parameters were investigated using simple linear regression analyses. A *p* value of < 0.05 indicated statistical significance. Adjusted R-squared (R^2^) values indicate variations (%) in parameters affecting a response variable. Variance inflation factors indicate the amount of multicollinearity in a set of regression variables. Furthermore, 95% confidence interval and prediction interval indicate uncertainty levels in statistical estimates. Statistical analyses were performed using SPSS (version 22.0; IBM Corp., Armonk, NY, USA).

### Ethics approval and consent to participate

It was a cross-sectional study, and the medical datasets were analyzed. Permission for accessing patients’ medical datasets was granted by the Director of the Center of Community Medicine, National Yang Ming Chiao Tung University Hospital, Yilan, Taiwan. All procedures performed in this study were in accordance with the ethical principles of the World Medical Association Declaration of Helsinki and National Yang Ming Chiao Tung University Hospital.

### Study approval statement

This study was reviewed and approved by the Human Subject Research Ethics Committee of National Yang Ming Chiao Tung University Hospital (approval number, 2020A012).

### Consent to participate statement

Written informed consent for participation was obtained from all participants.

## Results

**Figure 1:**
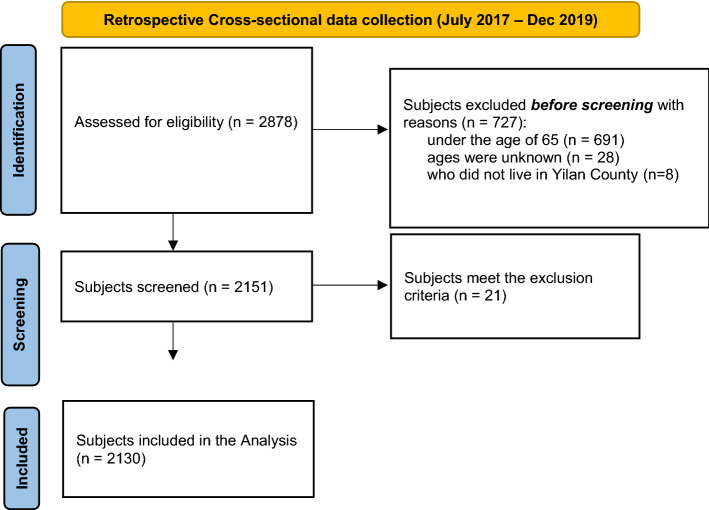
Flow chart depicting the number of individuals in our research that were included and excluded.

A total of 2,878 older adults were assessed for their eligibility to participate in the present study. This study excluded a total 691 individuals aged < 65 years, 28 whose ages were unknown, 8 who lived outside Yilan County, and 21 who met any one of the study exclusion criteria. Thus, a total of 748 people were excluded from this study; finally, a total of 2,130 people were included (Fig. 1). All physical fitness tests involved a total of 2,130 individuals aged ≥ 65 years (men, 547; mean age, 74.83 ± 6.54 years). The older adults were divided into six age groups: 65–69, 70–74, 75–79, 80–84, 85–89, and ≥ 90 years. The 65–69-year age group had the highest number of participants. The frailty questionnaire, which is used to assess recalled the average weekly activity levels in the previous month, revealed an average activity level of 13.74 ± 39.94 metabolic equivalent hours per week (men, 14.44 ± 44.92 MET-hours/week; women, 13.04 ± 38.07 MET-hours/week). Table 1 summarizes the participant demographics.

**Table 1:** Participant demographics (*N* = 2,130).

Item	n	%
**Gender**		
Female	1583	74.3
Male	547	25.7
**Age (years)**		
65–69	607	28.5
70–74	518	24.3
75–79	533	25
80–84	317	14.9
85–89	129	6
90 or older	26	1.2
**Physical activity level (MET-hour/week)**		
< 10	721	33.9
≧10, < 25	732	34.5
≧25	672	31.6

### Physical fitness levels of community-dwelling older adults in Taiwan

Table 2 presents the 5th, 10th, 25th, 50th, 75th, 90th, and 95th percentiles of the participants’ (stratified by age and sex) physical fitness levels, height and weight, sarcopenia results (e.g., HGS, timed 6-m walking speed test, and calf circumference), and bone density. These percentile values can be used to determine the fitness levels of active community-dwelling older adults in Taiwan with regard to their age and sex. Younger age was associated with improved performance.

**Table 2:** Percentile distribution of various physical fitness test results of community-dwelling older adults.

Handgrip strength test (kg)									
Age	*n*	*M* ± *SD*	*P*_5_	*P*_10_	*P*_25_	*P*_50_	*P*_75_	*P*_90_	*P*_95_
**Male**									
65–69	97	35.11 ± 0.80	21.80	26.06	30.00	35.60	40.05	44.60	48.67
70–74	133	33.31 ± 0.63	20.47	23.80	28.95	33.70	38.20	41.92	45.15
75–79	150	30.56 ± 0.60	18.93	21.33	25.35	29.50	35.35	39.96	41.99
80–84	111	29.13 ± 0.56	19.44	21.48	24.80	29.40	34.10	36.30	38.52
85–89	36	27.19 ± 1.09	17.43	19.24	21.85	27.75	32.30	37.50	38.65
90-older	18	24.27 ± 1.02	15.90	20.40	21.20	23.20	26.90	31.59	
**Female**									
65–69	457	23.95 ± 0.21	17.00	18.40	21.00	23.90	26.70	29.62	31.53
70–74	379	22.79 ± 0.23	15.40	17.00	19.90	23.00	25.90	27.90	30.10
75–79	391	21.33 ± 0.23	13.26	15.50	18.30	21.50	24.30	26.80	28.50
80–84	229	19.99 ± 0.27	13.25	14.80	17.30	20.10	22.45	24.70	26.95
85–89	110	17.63 ± 0.36	12.24	13.00	14.78	17.95	20.30	21.90	23.78
90-older	16	18.31 ± 0.87	12.50	12.85	15.28	20.30	21.33	21.86	

Height measurement (cm)									
Age	*n*	*M* ± *SD*	*P*_5_	*P*_10_	*P*_25_	*P*_50_	*P*_75_	*P*_90_	*P*_95_
**Male**									
65–69	97	163.36 ± 6.07	152.96	155.08	159.50	163.50	166.60	172.44	174.38
70–74	133	162.84 ± 5.76	152.00	155.30	159.70	163.00	165.90	170.20	173.70
75–79	150	161.32 ± 5.67	150.33	153.19	158.00	161.60	164.58	166.96	171.67
80–84	111	161.09 ± 5.89	151.15	154.12	157.00	161.00	165.05	168.66	170.56
85–89	37	161.36 ± 6.11	149.91	152.74	157.20	160.70	166.65	168.60	171.67
90-older	18	161.09 ± 4.45	155.50	155.74	158.80	162.00	163.65	168.78	
**Female**									
65–69	458	152.98 ± 5.49	143.60	146.00	149.20	153.00	156.10	159.53	162.01
70–74	379	152.27 ± 4.98	144.00	146.00	149.00	152.00	156.00	158.96	160.26
75–79	391	151.00 ± 5.28	142.32	144.00	147.20	151.00	154.10	158.00	160.00
80–84	229	149.60 ± 5.32	140.47	142.09	146.63	149.75	153.00	156.21	159.18
85–89	110	148.46 ± 5.99	137.11	140.26	144.38	148.10	151.08	154.72	156.66
90-older	16	146.83 ± 6.77	133.90	134.06	143.00	148.10	152.85	157.16	

Weight measurement (kg)									
Age	*n*	*M* ± *SD*	*P*_5_	*P*_10_	*P*_25_	*P*_50_	*P*_75_	*P*_90_	*P*_95_
**Male**									
65–69	97	68.45 ± 11.20	51.62	55.06	60.80	67.30	74.80	83.46	90.02
70–74	133	65.99 ± 9.66	50.54	53.02	59.40	65.90	73.00	78.86	84.36
75–79	150	65.12 ± 10.45	47.56	51.06	57.50	64.60	70.88	78.05	82.32
80–84	111	62.94 ± 8.78	49.13	52.52	56.85	62.50	68.55	74.98	79.53
85–89	37	63.99 ± 9.18	50.51	55.88	58.70	65.10	69.10	78.14	80.76
90-older	18	63.01 ± 6.45	54.10	54.18	57.35	63.20	65.40	75.70	
**Female**									
65–69	458	60.65 ± 10.57	46.27	48.87	53.45	59.00	65.80	75.10	80.48
70–74	379	59.69 ± 9.21	45.62	49.14	54.00	58.30	65.00	71.74	77.24
75–79	391	57.64 ± 9.01	43.30	46.28	51.00	57.30	63.80	68.98	72.88
80–84	229	56.36 ± 9.12	43.33	44.63	49.20	55.10	61.40	68.91	72.22
85–89	110	54.49 ± 10.81	40.90	42.24	47.25	52.50	58.60	69.33	76.08
90-older	16	50.43 ± 8.68	34.70	36.26	40.25	52.00	56.35	63.66	

Single-leg standing test (s)									
Age	*n*	*M* ± *SD*	*P*_5_	*P*_10_	*P*_25_	*P*_50_	*P*_75_	*P*_90_	*P*_95_
**Male**									
65–69	110	28.45 ± 1.70	3.99	5.26	13.92	30.00	414.10	42.10	43.49
70–74	133	23.47 ± 1.27	2.25	3.52	10.46	25.58	36.68	41.73	42.89
75–79	147	16.04 ± 1.08	1.87	2.40	5.03	11.61	30.00	37.74	40.81
80–84	103	11.84 ± 1.13	1.62	1.98	3.06	7.01	16.00	32.95	39.74
85–89	30	9.62 ± 1.95	0.55	1.31	2.49	4.76	14.05	29.99	36.33
90-older	11	3.57 ± .048	1.00	1.30	2.55	3.00	5.38	6.00	
**Female**									
65–69	494	25.55 ± 0.63	3.69	5.46	13.00	29.99	37.97	41.58	42.48
70–74	379	20.60 ± 0.70	2.00	3.89	8.16	18.00	30.00	40.94	41.53
75–79	375	14.33 ± 0.62	1.72	2.36	4.53	10.01	22.20	32.91	40.32
80–84	201	9.66 ± 0.69	1.33	1.85	3.12	6.00	12.06	22.06	35.93
85–89	88	5.69 ± 0.62	1.24	1.47	2.32	4.08	6.19	11.04	20.19
90-older	11	5.64 ± 2.16	1.28	1.32	1.50	3.70	4.59	22.64	

Chair sit-and-reach test (cm)*									
Age	*n*	*M* ± *SD*	*P*_5_	*P*_10_	*P*_25_	*P*_50_	*P*_75_	*P*_90_	*P*_95_
**Male**									
65–69	108	0.30 ± 0.96	− 14.80	− 12.41	− 7.95	0.15	7.73	12.47	18.17
70–74	133	1.23 ± 0.95	− 14.39	− 12.04	− 8.30	− 0.80	10.50	17.12	21.59
75–79	147	− 0.49 ± 0.71	− 14.20	− 11.14	− 7.30	− 1.20	4.80	10.54	15.16
80–84	98	− 1.95 ± 1.02	− 15.71	− 13.32	− 10.43	− 4.80	5.45	12.96	17.31
85–89	32	− 5.06 ± 1.88	− 20.97	− 17.04	− 15.00	− 6.30	4.13	7.61	14.60
90-older	10	− 5.36 ± 2.71	− 15.00	− 14.66	− 11.23	− 7.90	− 2.10	13.80	
**Female**									
65–69	493	8.26 ± .0.43	− 8.00	− 5.20	1.25	8.10	15.65	21.30	24.50
70–74	379	7.77 ± 0.47	− 7.90	− 3.90	1.10	7.50	14.51	21.56	24.10
75–79	382	6.27 ± .078	-10.29	-7.17	-1.83	6.00	13.43	18.70	20.19
80–84	209	4.55 ± 0.64	-12.30	-8.60	-1.55	4.10	11.30	16.80	19.65
85–89	96	2.63 ± .088	-9.00	-8.20	-3.38	0.85	8.78	14.13	17.83
90-older	13	1.78 ± 2.28	-10.50	-9.74	-5.10	1.80	8.75	14.50	

8-foot up-and-go (s)									
Age	*n*	*M* ± *SD*	*P*_5_	*P*_10_	*P*_25_	*P*_50_	*P*_75_	*P*_90_	*P*_95_
**Male**									
65–69	110	6.79 ± 0.21	9.77	8.61	7.44	6.32	5.66	5.11	4.94
70–74	135	7.63 ± 0.21	12.97	10.48	8.65	6.89	5.99	5.53	5.11
75–79	150	8.75 ± 0.23	14.03	12.12	9.69	8.22	6.76	6.19	5.68
80–84	105	9.41 ± 0.32	15.89	13.60	10.69	8.49	7.46	6.68	6.23
85–89	33	12.05 ± 0.80	22.37	19.28	14.87	11.41	8.55	6.95	6.69
90-older	13	11.28 ± 0.98		16.85	12.82	12.08	9.64	8.74	8.61
**Female**									
65–69	496	7.02 ± 0.09	9.89	8.92	7.64	6.64	5.93	5.34	5.04
70–74	383	7.69 ± 0.11	12.04	10.32	8.48	7.20	6.34	5.72	5.23
75–79	383	9.06 ± 0.21	14.30	12.49	9.95	8.10	6.93	5.25	5.81
80–84	212	10.33 ± 0.38	19.21	14.07	10.84	9.07	7.72	6.82	6.32
85–89	96	13.29 ± 0.65	26.73	21.46	15.30	11.18	9.36	7.97	7.17
90-older	13	12.80 ± 0.98		18.57	15.38	12.70	10.09	7.64	7.21

30-s chair stand test (number of repeats)									
Age	*n*	*M* ± *SD*	*P*_5_	*P*_10_	*P*_25_	*P*_50_	*P*_75_	*P*_90_	*P*_95_
**Male**									
65–69	110	18.7 ± 0.55	11.0	12.1	15.0	18.0	22.0	27.0	29.0
70–74	134	16.9 ± 0.46	8.0	10.0	13.0	17.0	20.0	25.0	25.3
75–79	150	15.7 ± 0.37	9.0	10.0	13.0	15.0	18.0	22.0	25.0
80–84	105	13.8 ± 0.44	6.0	8.0	10.5	14.0	17.0	20.0	21.0
85–89	31	12.2 ± 0.81	3.6	6.2	9.0	12.0	15.0	18.0	20.6
90-older	12	9.3 ± 0.58	6.0	6.0	8.3	9.0	10.8	12.4	
**Female**									
65–69	495	18.6 ± 0.22	11.0	12.0	15.0	18.0	22.0	25.0	28.0
70–74	382	16.7 ± 0.26	9.2	11.0	13.0	16.0	20.0	22.0	25.0
75–79	380	15.0 ± 0.23	8.0	10.0	12.0	15.0	18.0	21.0	23.0
80–84	208	13.9 ± 0.28	7.0	9.0	11.0	14.0	16.0	19.0	21.0
85–89	95	11.0 ± 0.48	4.8	5.0	8.0	11.0	15.0	17.4	18.2
90-older	13	8.5 ± 0.75	3.0	4.2	7.0	8.0	11.0	12.2	

2-min step test (number of steps)									
Age	*n*	*M* ± *SD*	*P*_5_	*P*_10_	*P*_25_	*P*_50_	*P*_75_	*P*_90_	*P*_95_
**Male**									
65–69	109	115.9 ± 1.75	89.5	93.0	103.5	116.0	127.0	137.0	143.5
70–74	133	111.2 ± 1.44	83.0	91.4	102.0	111.0	120.0	132.6	139.3
75–79	147	105.0 ± 1.58	77.4	83.0	95.0	105.0	119.0	128.2	134.6
80–84	98	103.6 ± 1.82	78.9	82.8	94.8	104.0	114.0	122.1	125.0
85–89	30	89.2 ± 4.83	24.8	51.6	76.5	93.5	103.3	117.9	128.6
90 and older	11	89.0 ± 7.46	37.0	40.6	74.0	98.0	107.0	114.8	
**Female**									
65–69	492	116.4 ± 0.83	89.7	97.0	108.0	117.0	127.0	136.0	145.0
70–74	373	111.6 ± 0.91	86.0	94.0	103.0	112.0	121.0	132.0	139.0
75–79	372	105.3 ± 1.08	68.7	81.0	95.0	107.0	118.0	128.7	133.0
80–84	203	101.8 ± 1.40	65.2	77.8	93.0	104.0	113.0	122.0	128.6
85–89	88	95.7 ± 2.81	40.9	63.5	88.3	99.5	108.8	120.1	135.7
90-older	12	89.6 ± 8.99		18.9	87.8	100.5	103.5	113.6	

30-s arm curl test (number of repeats)									
Age	*n*	*M* ± *SD*	*P*_5_	*P*_10_	*P*_25_	*P*_50_	*P*_75_	*P*_90_	*P*_95_
**Male**									
65–69	109	20.6 ± 0.41	15.0	16.0	17.0	20.0	24.0	27.0	27.5
70–74	135	19.0 ± 0.37	12.0	13.6	18.0	19.0	22.0	24.0	26.0
75–79	150	17.2 ± 0.33	10.0	13.0	14.8	17.0	20.0	22.0	23.5
80–84	104	16.4 ± 0.45	8.0	11.0	13.0	17.0	19.0	22.0	25.0
85–89	32	14.6 ± 0.77	6.0	8.9	12.0	14.0	16.0	22.1	24.0
90-older	13	12.2 ± 0.64	9.0	9.0	10.5	12.0	13.5	16.2	
**Female**									
65–69	495	20.5 ± 0.18	15.0	16.0	18.0	20.0	23.0	26.0	27.0
70–74	382	19.1 ± 0.21	13.0	15.0	16.0	19.0	22.0	24.7	26.0
75–79	381	17.6 ± 0.22	11.0	12.0	15.0	18.0	21.0	23.0	25.0
80–84	210	16.6 ± 0.28	10.0	12.0	14.0	17.0	19.0	22.0	23.0
85–89	94	14.0 ± 0.50	5.8	7.0	12.0	14.0	17.0	19.5	22.0
90-older	13	13.5 ± 1.09	5.0	5.8	12.0	14.0	15.0	18.8	

Timed 6-m walking speed test (s)									
Age	*n*	*M* ± *SD*	*P*_5_	*P*_10_	*P*_25_	*P*_50_	*P*_75_	*P*_90_	*P*_95_
**Male**									
65–69	110	4.82 ± 0.15	6.34	5.88	5.22	4.57	4.00	3.68	3.36
70–74	135	4.96 ± 0.11	7.42	6.62	5.40	4.80	4.16	3.73	3.49
75–79	150	5.56 ± 0.12	8.59	7.52	6.17	5.29	4.52	4.10	3.79
80–84	104	6.14 ± 0.19	10.40	8.38	6.80	5.64	5.00	4.51	4.03
85–89	33	7.40 ± 0.37	11.99	10.34	8.91	7.26	5.65	4.75	4.51
90-older	13	7.76 ± 0.47		10.36	9.26	7.60	6.09	5.68	5.50
**Female**									
65–69	496	4.81 ± 0.05	6.38	5.99	5.26	4.62	4.10	3.84	3.56
70–74	383	5.29 ± 0.07	7.38	6.85	5.76	5.10	4.50	4.00	3.71
75–79	382	6.06 ± 0.14	9.95	7.87	6.54	5.59	4.82	4.25	4.00
80–84	212	6.85 ± 0.28	13.44	8.86	7.00	5.98	5.00	4.67	4.35
85–89	96	8.51 ± 0.49	16.90	14.88	9.35	6.99	5.74	5.30	4.52
90-older	13	7.50 ± 0.52		10.36	9.00	7.64	5.75	4.94	4.88

Back scratch test (cm)**									
Age	*n*	*M* ± *SD*	*P*_5_	*P*_10_	*P*_25_	*P*_50_	*P*_75_	*P*_90_	*P*_95_
**Male**									
65–69	106	− 14.28 ± 1.27	− 35.30	− 32.00	− 25.00	− 14.50	− 0.75	3.00	4.00
70–74	134	− 14.28 ± 1.21	− 36.00	− 32.50	− 25.00	− 15.00	− 0.88	4.00	7.00
75–79	144	− 16.75 ± 1.13	− 36.75	− 34.50	− 27.38	− 17.00	− 7.63	1.00	3.38
80–84	98	− 16.46 ± 1.38	− 41.00	− 32.30	− 24.25	− 17.00	− 9.75	3.00	6.10
85–89	32	− 18.48 ± 2.72	− 44.35	− 43.40	− 33.50	− 14.00	− 6.00	− 0.30	3.75
90-older	12	− 24.54 ± 2.41	− 35.00	− 35.00	− 34.38	− 21.75	− 20.00	− 11.85	
**Female**									
65–69	488	− 3.98 ± 0.47	− 26.00	− 18.00	− 10.00	0.00	3.50	6.05	8.00
70–74	377	− 6.44 ± 0.61	− 28.05	− 23.60	− 15.00	− 2.00	3.00	5.50	8.00
75–79	371	− 7.95 ± 0.66	− 30.00	− 25.80	− 18.00	− 5.00	2.50	5.00	7.00
80–84	204	− 11.07 ± 0.82	− 31.88	− 25.75	− 20.00	− 10.25	0.00	3.00	4.88
85–89	89	− 13.99 ± 1.36	− 32.00	− 30.00	− 24.00	− 15.00	− 3.75	4.00	7.00
90-older	13	− 19.08 ± 4.28	− 45.00	− 41.40	− 30.50	− 17.00	− 8.25	5.30	

Calf circumference measurement (cm)									
Age	*n*	*M* ± *SD*	*P*_5_	*P*_10_	*P*_25_	*P*_50_	*P*_75_	*P*_90_	*P*_95_
**Male**									
65–69	62	35.8 ± 0.40	30.4	31.3	33.4	35.9	37.8	39.9	41.3
70–74	62	35.4 ± 0.37	30.2	32.0	33.7	35.3	37.0	39.0	40.2
75–79	66	33.8 ± 0.34	29.0	30.0	32.1	33.6	35.6	37.4	39.3
80–84	51	33.3 ± 0.38	28.3	29.6	31.3	33.5	35.2	36.5	37.7
85–89	20	34.0 ± 0.57	28.0	31.3	32.5	34.0	36.4	37.5	38.6
90-older	8	31.7 ± 0.95	26.5	26.5	29.9	32.6	33.8		
**Female**									
65–69	228	34.7 ± 0.19	30.2	31.0	32.5	34.5	36.1	38.6	41.0
70–74	177	34.3 ± 0.24	29.5	30.1	32.1	34.1	36.3	38.3	39.5
75–79	187	33.6 ± 0.23	28.7	29.4	31.5	33.2	35.5	38.0	39.3
80–84	111	35.9 ± 3.02	28.0	29.0	30.1	32.5	35.3	37.4	39.1
85–89	42	31.4 ± 0.54	26.5	27.7	29.1	30.7	33.5	36.4	39.1
90-older	6	29.8 ± 1.17	26.1	26.1	27.4	29.5	32.8		

Bone mass density measurement (T-score)									
Age	*n*	*M* ± *SD*	*P*_5_	*P*_10_	*P*_25_	*P*_50_	*P*_75_	*P*_90_	*P*_95_
**Male**									
65–69	62	− 0.80 ± 0.15	− 2.78	− 2.51	− 1.60	− 0.75	0.23	0.64	0.91
70–74	62	− 0.96 ± 0.14	− 2.84	− 2.49	− 1.78	− 0.80	− 0.28	0.50	0.69
75–79	66	− 1.17 ± 0.16	− 3.51	− 2.94	− 1.99	− 1.30	− 0.17	0.76	1.30
80–84	51	− 1.27 ± 0.17	− 3.06	− 2.68	− 2.20	− 1.50	− 0.41	0.72	1.00
85–89	20	− 1.24 ± 0.24	− 2.84	− 2.55	− 1.92	− 1.44	− 0.61	0.42	1.47
90-older	8	− 1.59 ± 0.63	− 4.23	− 4.23	− 2.64	− 2.29	0.43		
**Female**									
65–69	228	− 0.72 ± 0.08	− 2.60	− 2.20	− 1.53	− 0.78	0.00	1.00	1.56
70–74	177	− 0.94 ± 0.09	− 2.83	− 2.42	− 1.82	− 1.10	− 0.20	0.82	1.31
75–79	187	− 1.36 ± 0.08	− 3.18	− 2.98	− 2.19	− 1.35	− 0.50	0.10	0.49
80–84	111	− 1.38 ± 0.10	− 3.12	− 2.84	− 2.10	− 1.40	− 0.73	0.28	0.80
85–89	42	− 1.66 ± 0.15	− 3.40	− 2.67	− 2.28	− 1.70	− 1.07	− 0.43	0.04
90-older	6	− 1.85 ± 0.35	− 2.87	− 2.87	− 2.70	− 1.90	− 1.00		

Body mass index measurement (kg/m^2^)									
Age	*n*	*M* ± *SD*	*P*_5_	*P*_10_	*P*_25_	*P*_50_	*P*_75_	*P*_90_	*P*_95_
**Male**									
65–69	97	25.61 ± 3.64	19.93	20.488	23.33	25.15	27.15	31.05	32.71
70–74	133	24.86 ± 3.19	19.84	20.70	22.63	25.02	27.10	29.65	30.55
75–79	150	24.98 ± 3.56	19.01	20.34	22.36	24.59	26.82	28.98	30.77
80–84	111	24.24 ± 2.99	18.54	20.63	22.38	24.40	25.96	27.88	29.37
85–89	37	24.53 ± 2.91	20.06	21.46	23.35	24.87	26.56	27.95	29.17
90-older	18	24.36 ± 3.01	19.39	19.70	20.85	24.23	26.02	29.91	
**Female**									
65–69	458	25.89 ± 4.07	19.87	21.13	23.11	25.35	28.23	31.43	33.46
70–74	374	25.73 ± 3.68	20.40	21.37	23.19	25.28	28.03	30.18	32.58
75–79	391	25.28 ± 3.74	19.52	20.48	22.66	25.19	27.61	30.22	31.84
80–84	229	25.17 ± 3.76	19.43	20.34	22.25	24.82	27.45	29.99	32.11
85–89	110	24.69 ± 4.52	18.52	19.87	21.78	23.79	26.84	30.47	32.85
90-older	16	23.32 ± 3.27	17.04	17.92	19.68	22.93	25.42	27.16	

*A negative score indicates that the fingertip does not pass the 0 mark.

**A negative score indicates that the fingertips do not overlap.

Among men, older adults aged 65–69 years had the highest BMI (25.61 ± 3.64 kg/m^2^), whereas those aged 80–84 years had the lowest BMI (24.24 ± 2.99 kg/m^2^). Regarding the single-leg standing test, the best results were noted in older adults aged 65–69 years (28.45 ± 1.70 s) and the worst results were noted in those aged ≥ 90 years (3.57 ± 0.48 s). Older adults aged 65–69 and 70–74 years exhibited the best results in the back scratch (− 14.28 ± 1.27 cm) and chair sit-and-reach (1.23 ± 0.95 cm) tests, respectively; however, older adults aged ≥ 90 years had the poorest performance in both tests (− 24.54 ± 2.41 and − 5.36 ± 2.71 cm, respectively). In 30-s arm curl and 30-s chair stand tests, older adults aged 65–69 years exhibited the best performance (20.6 ± 0.41 and 18.7 ± 0.55 repeats, respectively), whereas those aged ≥ 90 years exhibited the worst performance (12.2 ± 0.64 and 9.3 ± 0.58 repeats, respectively). Moreover, older adults aged 85–89 years took the longest time to complete 8-foot up-and-go test (12.05 ± 0.80 s), whereas those aged 65–69 years took the least time (6.79 ± 0.21 s). In the 2-min step test, older adults aged 65–69 years exhibited the highest performance (115.9 ± 1.75 steps), whereas those aged ≥ 90 years exhibited the lowest performance (89.0 ± 7.46 steps). Furthermore, in HGS test, older adults aged 65–69 years had the highest strength (35.11 ± 0.80 kg), whereas those aged ≥ 90 years had the lowest strength (24.27 ± 1.02 kg). The highest calf circumference (35.8 ± 0.40 cm) was observed among older adults aged 65–69 years, whereas the lowest calf circumference (31.7 ± 0.95 cm) was noted among those aged ≥ 90 years. In the timed 6-m walking speed test, older adults aged 65–69 years had the best performance (4.82 ± 0.15 s), whereas those aged ≥ 90 years had the worst performance (7.76 ± 0.47 s). In addition, physical performance was poorer (gait speed < 1.0 m/s) in older adults aged 80–84, 85–89, and ≥ 90 years than in the other age groups. Among men, bone density decreased with age (measured as the T-score; decreased from − 0.80 ± 0.15 to − 1.59 ± 0.63). Table 2 summarizes the test results of older men.

Among women, BMI was the highest in older adults aged 65–69 years (25.89 ± 4.07 kg/m^2^) and the lowest those aged ≥ 90 years (23.32 ± 3.27 kg/m^2^). In the single-leg standing test, older adults aged 65–69 years exhibited the best performance (25.55 ± 0.63 s), whereas those aged ≥ 90 years exhibited the worst performance (5.64 ± 2.16 s). In back scratch and chair sit-and-reach tests, best performance levels were noted in older adults aged 65–69 years (− 3.98 ± 0.47 and 8.26 ± 0.43 cm, respectively), whereas the worst performance was noted in those aged ≥ 90 years. Moreover, in 30-s arm curl and 30-s chair stand tests, older adults aged 65–69 years had the best performance (20.5 ± 0.18 and 18.6 ± 0.22 repeats, respectively), whereas those aged ≥ 90 years had the worst performance (13.5 ± 1.09 and 8.5 ± 0.75 repeats, respectively). Older adults aged 85–89 years took the longest time to complete 8-foot up-and-go test (13.29 ± 0.65 s), whereas those aged 65–69 years took the least time (7.02 ± 0.09 s). In the 2-min step test, older adults aged 65–69 years exhibited the best performance (116.4 ± 0.83 steps), whereas those aged ≥ 90 years exhibited the worst performance (89.6 ± 8.99 steps). In HGS, older adults aged 65–69 years had the highest strength (23.95 ± 0.21 kg), whereas those aged 85–89 years had the lowest strength (17.63 ± 0.36 kg). Calf circumference exhibited a tendency to decrease with age; older adults aged ≥ 90 had the lowest calf circumference (29.8 ± 1.17 cm). In the timed 6-m walking speed test, older adults aged 65–69 years had the best results (4.81 ± 0.05 s) whereas those aged 85–89 years had the worst results (8.51 ± 0.49 s). Slow gait speed was associated with poor physical performance in older adults aged 75–79, 80–84, 85–89, and ≥ 90 years. Bone density (T-score) decreased with age (from − 0.72 ± 0.08 to − 1.85 ± 0.35). Table 2 summarizes the test results of older women.

### HGS analyzed using a stepwise multiple linear regression model in older adults

We performed stepwise regression analysis to evaluate the changes (R^2^) in the result of each physical fitness test. High adjusted R^2^ values suggest that the regression model can predict the dependent variable and also explain the variance in the response variable around its mean. Thus, we constructed an HGS model; high R^2^ values obtained using this model indicated that the results of the stepwise regression analysis fit well with our data. For community-dwelling older adults, 59.3% of the variance in HGS equations could be explained by age; sex; BMI; and the results of 30-s arm curl, 8-foot up-and-go, and back scratch tests. The HGS model for older adults was as follows: HGS = 24.688 + (11.954 × sex) + (0.294 × 30-s arm curl test result) – (0.548 × 8-foot up-and-go test result) – (0.12 × age) + (0.222 × BMI) + (0.036 × back scratch test result). The 95% prediction interval for this estimate was approximately ± 9.28. As indicated in Table 3, no evidence of multicollinearity was noted among the predictive factors, such as age, sex, BMI, and the results of 30-s arm curl, 8-foot up-and-go, and back scratch tests. The stepwise regression analysis revealed that HGS had a negative correlation with age and 8-foot up-and-go test result (*p* < 0.05) but a positive correlation with sex (*p* < 0.05), BMI (*p* < 0.05), and back scratch test result (*p* < 0.05). Furthermore, the variance inflation factors were all < 5, which indicated that the predictors were not correlated.

**Table 3:** Handgrip strength analyzed using a stepwise multiple linear regression model.

Variable	Unstandardized coefficients		Standardized coefficients	t	Sig	95.0% confidence interval for B		Collinearity statistics	
	B	SE	Beta			Lower bound	Upper bound	Tolerance	VIF
6 (Constant)	24.688	2.781		8.877	0.000	19.229	30.146		
Gender (male:1, female:0)	11.954	0.393	0.705	30.448	0.000	11.183	12.724	0.837	1.195
30-s arm curl	0.294	0.046	0.169	6.353	0.000	0.203	0.385	0.637	1.571
8-feet up-and-go	− .0.548	0.100	− .0.156	− 5.465	0.000	− .0.745	− 0.351	0.547	1.827
Age	− 0.120	0.029	− 0.104	− 4.096	0.000	− .0.178	− .0.063	0.689	1.451
BMI	0.222	0.045	0.112	4.897	0.000	0.133	0.311	0.857	1.167
Back scratch	0.036	0.014	0.066	2.658	0.008	0.009	0.063	0.720	1.389

*N* = 2,130; adjusted R-square, 0.593.

### HGS analyzed separately for men and women through stepwise multiple linear analysis

Sex was strongly correlated with HGS, and the R^2^ value of the total population corroborated this result. Table 4 presents the results of stepwise regression analysis with the data of participants stratified by sex. The HGS equation for older men was as follows: HGS = 19.973 + (0.754 × calf circumference) + (0.318 × 30-s chair stand test result) − (0.827 × 8-foot up-and-go test result) − (0.151 × age). The factors in this equation were calf circumference, age, and the results of 30-s chair stand and 8-foot up-and-go tests (adjusted R^2^: male, 0.36). By contrast, the HGS equation for older women was as follows: HGS = 27.489 + (0.305 × 30-s arm curl test result) − (0.495 × 8-foot up-and-go test result) – (0.098 × age) + (0.138 × BMI) + (0.032 × back scratch test result). The factors in this equation were age; BMI; and 30-s arm curl, 8-foot up-and-go, and back scratch results (adjusted R^2^: women, 0.271).

**Table 4:** Handgrip strength analyzed separately for men and women through stepwise multiple linear regression analysis.

Variable	Male (n = 547)		t	Sig	Female (n = 1,583)		t	Sig
	Unstandardized coefficients	Standardized coefficients			Unstandardized coefficients	Standardized coefficients		
	B	Beta			B	Beta		
(Constant)	19.973		2.334	0.020	27.489		8.841	0.000
8-feet up-and-go	− .0.827	− 0.215	− 2.727	0.007	− 0.495	− .0.230	− 5.084	0.000
Calf circumference	0.754	0.296	5.347	0.000				
30-s chair stand	0.318	0.216	2.906	0.004				
Age	− 0.151	− 0.132	− 2.078	0.039	− 0.098	− 0.134	− 3.307	0.001
30-s arm curl					0.305	0.274	6.343	0.000
Back scratch					0.032	0.086	2.308	0.021
BMI					0.138	0.116	3.217	0.001

Adjusted R-square: men, 0.36; women, 0.271.

### Results of the simple linear regression with age as the predictor variable and physical fitness parameters as the dependent variables

All physical fitness parameters declined with age in both older men and women. A strong negative correlation was noted between age and HGS. With increasing age, the HGS of older men and women decreased by 0.437 and 0.289 kg every year, respectively. The decrease in HGS with age was more rapid in men than in women. Furthermore, with increasing aging, bone density and the results of 30-s chair stand, 2-min step, and back scratch tests were less favorable and timed 6-m walking was more favorable in women than in men. Table 5 presents the correlation between age and physical fitness parameters.

**Table 5:** Results of the simple linear regression analysis performed using age as the predictor variable and physical fitness parameters as the dependent variables.

	Male (n = 547)				Female (n = 1583)			
	R square	Unstandardized coefficients B	Standardized coefficients beta	sig	R square	Unstandardized coefficients B	Standardized coefficients beta	Sig
Hand grip strength (kg)	0.149	− 0.437	− .0.386	0.000	0.157	− 0.289	− 0.396	0.000
Single-leg standing (sec)	0.192	− 1.045	− 0.438	0.000	0.217	− 1.034	− 0.466	0.000
Chair sit-and-reach (cm)	0.021	− 0.221	− 0.144	0.001	0.036	− 0.282	− 0.191	0.000
8-feet up-and-go (sec)	0.175	0.200	0.418	0.000	0.261	0.177	0.420	0.000
30-s chair stand (number of repeats)	0.152	− 0.318	− 0.390	0.000	0.191	− 0.350	− 0.437	0.000
2-min step (number of steps)	0.113	− 1.027	− 0.336	0.000	0.111	− 1.076	− 0.333	0.000
30-s arm curl (number of repeats)	0.169	− 0.290	− 0.411	0.000	0.178	− 0.297	− 0.422	0.000
Timed 6-m walk (sec)	0.161	0.106	0.401	0.000	0.135	0.153	0.367	0.000
Back scratch (cm)	0.017	− 0.268	− 0.129	0.003	0.063	− 0.469	− 0.250	0.000
Calf circumference (cm)	0.115	− 0.146	− 0.339	0.000	0.078	− 0.145	− 0.280	0.000
Bone mass density (T-score)	0.027	− 0.029	− 0.164	0.007	0.075	− 0.052	− 0.274	0.000

## Discussion

We evaluated the physical fitness levels of a rural community-dwelling older adult population in Taiwan and investigated the predictive factors for sarcopenia and osteoporosis. Although several studies have been conducted to assess the fitness levels of older adults across the globe^27,28,30–32,44^, only one study focused on the older adults in Taiwan^33^. However, in the aforementioned study conducted in Taiwan, the participants were not active community-dwelling older adults. Furthermore, in most studies, only a few aspects of physical fitness have been evaluated^22–26,28–33,44^; thus, the findings might not have reflected the full potential of older adults to safely engage in mundane daily physical activities without requiring assistance or becoming tired. The present study was conducted to devise strategies for the comprehensive long-term care of older adults. To the best of our knowledge, this study is the first to explore all aspects of physical fitness in active community-dwelling older adults and use a battery of widely recognized and acceptable physical fitness tests^27^ in addition to demographic and frailty surveys. The reference values for physical fitness were age-specific (5-year span) and sex-specific, which increases their clinical applicability. Our findings related to the percentile distribution of each physical fitness test result may be useful for physicians to design tailored programs for their patients on the basis of the patients’ physical fitness levels. These findings aid the development of various health promotion programs for active community-dwelling older adults; these individuals can also compare their physical performance levels with the standard for their age group. According to a study conducted > 10 years ago^33^, reference values for physical fitness must be updated, particularly for older adults who participate in community activities. Furthermore, physical performance reportedly worsens with age, which suggests that more precise models are needed to evaluate the performance levels of adults aged > 50 years^44^. The high variability and low explained variance of previous predictive models for older adults suggest that these estimates of the physical tests are not completely reliable. In the present study, HGS test was used as a simple and an effective assessment tool because the test can be completed rapidly and it helps obtain valuable information regarding the health status of older adults. Thus, we developed the aforementioned HGS equations on the basis of the stepwise regression analysis results; our finding may improve the understanding of physicians regarding the correlation between HGS and complete physical performance. Furthermore, on the basis of our study, various therapeutic strategies and educational programs may be developed to prevent or delay disability and promote health in community-dwelling older adults, thus reducing the economic burden associated with the treatment and hospitalization of these individuals.

As mentioned, the HGS test is a widely used, convenient tool for physical fitness assessment. Low HGS has been associated with physical limitations in individuals aged ≥ 60 years^35^ and also mortality^36^. HGS is a clinically important indicator of the health status of older adults. Therefore, it can be used as a substitute for other complex and time-consuming tests to evaluate the overall status of community-dwelling older adults. As mentioned, our model revealed that HGS is positively correlated with sex; BMI; and the results of 30-s arm curl, 8-foot up-and-go, and back scratch tests. These findings are consistent with those of a study reporting an association between HGS and BMI among Greek women^45^. By contrast, we found a negative correlation between age and HGS. Similar findings have been reported for other ethnicities^25,26,44–47^. HGS is a parameter of physical fitness and may indicate the physiological health status of an individual. The correlation of HGS with other commonly used physical fitness parameters, as shown in the present study, suggests that the associated physical fitness parameters can decline with decreasing HGS. Thus, our model represents an age- and sex-specific reference system for evaluating the efficacy of health promotion programs for community-dwelling older adults. Although the physical activity levels may influence the HGS of older adults, the stepwise process did not include them in the final regression model statistically. Physical activity differs from exercise. In the present study, the participants engaged in various activities in the community, such as calisthenics, strolling, and Qigong; however, their physical activity was neither intense nor sufficient for improving their overall strength. Therefore, well-designed programs for effectively improving the physical fitness of community-dwelling older adults are needed. Furthermore, in the domain of physical rehabilitation, the specific adaptation to imposed demands (SAID) principle indicates that the human body adapts specifically to imposed demands. If someone simply performs pull-up exercise using a pull-up bar, the body adapts to this unique physical effort but not necessarily to other climbing patterns or settings. To substantially improve HGS, exercise regimens must be designed according to the SAID principle.

In the present study, the strongest correlation was noted between HGS and sex. Although HGS was correlated with the results of 30-s chair stand test in men, it was correlated with the results of 30-s arm curl test in women. Notably, a study including American college students reported that female students exhibited 37%–68% of the muscular strength generally noted in men; compared with male students, female students exhibited high muscle strength in the upper body but low muscle strength in the lower body. The lower limbs of women are often stronger than their upper limbs and shoulders^48^. In the present study, we used the 30-s chair stand and 30-s arm curl tests to evaluate the strength/endurance of lower and upper extremities, respectively. Contrasting findings were noted in our study, and these findings might be due to the adaptive behaviors of the participants; specifically, in Yilan County, men are generally involved in farming and labor work that requires more crouching and sit-to-stand movements, whereas women are generally engaged in household chores and crafting (handicrafts). As shown in Table 5, the result of 30-s chair stand test decreases by 0.318 and 0.350 with 1-year increase in age among both men and women, respectively. Because the lower limb strength of women reduces faster than that of men, HGS may be more directly correlated with the results of 30-s arm curl test in women than in men.

The findings of the present study indicate that levels of performance in all physical fitness tests decline with aging in a consistent and steady manner in both men and women. We compared the results obtained using the HGS model with those of a study conducted in Taiwan. Adults with a BMI of < 18.5 kg/m^2^ are considered to be underweight by the Taiwanese Ministry of Health and Welfare; this low value may suggest malnutrition, eating disorders, or other health concerns. By contrast, adults with a BMI of ≥ 24 kg/m^2^ are considered to be overweight. In the present study, the BMI values of men and women were 24.24–25.61 and 23.32–25.89 kg/m^2^, respectively. Almost all older men and women were overweight, except women aged ≥ 90 years whose BMI was within the normal range (23.32 ± 3.27). A study conducted in Taiwan^33^ reported a BMI of 23.3–25.2 and 23.0–25.0 kg/m^2^ in men and women, respectively. In their study, men aged 60–64 years and women aged 65–69 years were overweight. We demonstrated that older adults who participate in community activities tend to have a high BMI. A study published in *The Lancet* in 2009, including a total of 900,000 adults, reported that individuals with underweight and overweight exhibit higher risks of mortality than do those with normal weight (determined using BMI)^49^. Despite good health and a low percentage of body fat, some people may be regarded as overweight on the basis of their BMI. Our findings indicate that many community-dwelling older adults have body composition–related problems, even if they participate in community activities. Thus, further studies on the distribution of muscle and fat mass are warranted.

In the present study, the 8-foot up-and-go test was used to assess agility and dynamic balance and determine the differences between recurrent faller and nonfaller groups used in a previous study^50^. Fallers are defined as older adults who take ≥ 8.5 s to complete the test (overall prediction rate, 82%). This test has a sensitivity of 78% and a specificity of 86%^50^. In the present study, men and women aged > 75 years took > 8.5 s; this highlights importance of fall prevention programs for this age group. Although the percentiles of all physical fitness scores of older adults stratified by age and sex were not reported in a study conducted in Taiwan^34^, their participants (both men and women) exhibited lower performance levels in 8-foot up-and-go and 30-s arm curl tests than did our participants. This difference may be explained by the inherent differences between the two older adult populations. In the aforementioned study, the participants were recruited through preannouncement at local communities by health-care facilities, clubs, and sports centers^34^. In the present study, the participants were older adults who engaged in community activities, such as calisthenics and walking, which might have improved their performance. Regarding the results of the flexibility test, older adults are less flexible than their younger counterparts. Women tend to be more flexible than their same-aged male counterparts. This is reasonable because of the metabolic changes occurring in the muscles; in addition, the loss of mitochondrial DNA may exert negative effects on the overall fitness levels of older adults^51^.

Our study has some limitations. First, this study included community-dwelling older adults who were functionally independent and engaged in community activities; the participants were the residents of a rural area in Taiwan. Thus, our findings may not be generalized beyond the study population because our data may not represent normative data. Normative data can be obtained by conducting studies involving a large and randomly selected representative sample from a wide population. Age, sex, and ethnicity should all be considered when defining the reference population. Moreover, older adults who do not participate in community activities must also be assessed. Online surveys may be conducted to collect normative data—something we wish to explore in our future studies given the suitability of our test model. Second, women outnumbered men in the present study. Similarly, in a study on the fitness levels of older people residing in the United States, the number of female participants was higher than that of male participants (men, 2,135; women, 5,048). By contrast, in a similar study conducted in Taiwan, the number of men was higher than that of women. This difference between the participation of men and women may be attributed to cultural and geographical factors; however, further studies are needed. Owing to the sex-specific differences, the sample size of men in our study (*n* = 547) might not have been adequate. Third, the cross-sectional design of the present study precluded causal inference. To investigate the possible causal relationship between HGS and physical fitness, a longitudinal design or qualitative methodology must be used in future studies. Finally, several researchers conducted the physical fitness tests and managed (i.e., arrangement, input, and evaluation) the obtained data. Thus, human error might have occurred. Nevertheless, to reduce data collection–related variability and enhance test efficiency and accuracy, we used a sensor-assisted physical fitness test system to evaluate the physical fitness of the participants.

## Conclusions

The age- and sex-specific reference values for physical fitness may facilitate the interpretation of physical fitness test results. These values can be used by health-care professionals to warn their patients regarding any risk of functional decline. In addition, the reference values can be used to design effective exercise programs for community-dwelling older adults. The stepwise regression analysis indicated that young men with high BMI values and high levels of performance in the 8-foot up-and-go and back scratch tests exhibit high HGS. Furthermore, the sex-specific analysis suggested that HGS is associated with upper and lower limb strength in women and men, respectively. Thus, our model and reference values may be useful for designing tailored programs for promoting health in older adults.

## Data Availability

The data that support the findings of this study are not publicly available because their containing information that could compromise the privacy of research participants but are available from the corresponding author upon reasonable request.
